# Dr. Adams, on the Digestion of the Stomach after Death, in Answer to Mr. Burns

**Published:** 1810-05

**Authors:** 


### Dr. Adams, *on the Digestion of the Stomach after Death, in Answer to Mr.* Burns.

NOTHING can be more agreeable to one who wishes to be understood, than to meet with objections, the answering which may tend to elucidate his opinions. It has often been remarked of polemics, that if two men, who mean well, could converse together for half an hour, however different they might fancy each other's sentiments, they would part satisfied with one another. If such is the case with controversies depending on the interpretation of words, how much more probable must it be where the whole depends on demonstrable facts.

Before entering on the particular passages of Mr. Burn's paper, in the last Number of the Edinburgh Journal, in which he has done me the honour to introduce my name, permit me to premise a few propositions, which should always be kept in view, while we attend to the digestion of the stomach after death.

1st. That the gastric juice is secreted in the stomach, in which cavity it reduces all digestible substances to a pultaceous mass.

2d. That the gastric juice does not, in the usual condition of the living body, digest the stomach itself.

3d. That after death certain parts of the stomach are sometimes found digested, or reduced to a mass similar to those substances which are affected by the gastric juice.

From these propositions Mr. Hunter first discovered that the property of this juice is to dissolve animal substance only when dead, which is further proved by the number of animals that live and grow in the stomachs of other animals without injury, but which, when dead, are soluble in the gastric juice of the same stomachs.

Spallanzani, with a patience that almost wearies his reader, made many attempts at dissolving the stomach by its own juice, but succeeded satisfactorily in none. He proved however however two things, viz. that the process of digestion continues after death, and that the stomach is digestible. The first by introducing food into the stomach after he had killed his animal; and the second by giving the stomach of one dog to be devoured by another. In both cases he found digestion take place. This convinced him of two leading facts, which led him to admit the probability of Mr. Hunter's, though he never could produce them.

The following passage is extracted from Mr. Hunter's paper,* to show, amongst other things, the propriety of his remarks on the mode of conducting experiments, and also the difficulty of drawing just conclusions from their result, until we have detected every fact with its series and order.

"Spallanzani (says Mr. Hunter) made some experiments, to prove that digestion is carried on after death; but they are not so conducted as to correspond with the appearances in the dead body. An experiment, although it may be very well and accurately made, so far as the experiment goes, if it does not preserve a close connection with the purpose for which it was made, the conclusions to be drawn from it cannot correspond with the intention. This is exactly the case with the experiments of Spallanzani, which although they prove that meat was digested in the stomach after the animal was killed, (which no one doubted) yet are not at all calculated to show that the stomach itself may be digested. In fact, the mode in which they were managed, rather tended to prevent that effect from taking place, the gastric juice having substances introduced on which it could act, was less likely to affect the coats of the stomach. That the digestion was not carried on merely by the gastric juice secreted before death, is evident from his own account, some of the food which had been introduced and digested, being found in the duodenum; a thing that could not have happened, if a cessation of the actions of life in the involuntary parts had taken place when visible life terminated. There had been an action, and most probably a secretion, in the stomach. The only experiment that can be made with any probability of a decided result is, to kill the animal while the stomach is empty, and observe what afterwards takes place. There are very few stomachs that have not, when examined after death, some of the inner villous coat destroyed;

---

* Observations on certain Parts of the Animal Oeconomy, p. 181.

stroyed; which may have been done by the gastric juice in the ducts of the glands which secrete it."

I have always thought Spallanzani, in one particular, unfairly treated in this passage; for it is certain that the case which first attracted Mr. Hunter's attention, and which he relates with most minuteness, was of a man, who just before the accident by which his skull was fractured, "had made a hearty supper of bread, cheese, and ale." Upon opening the abdomen, he adds, "the stomach, tho' it still contained a good deal, was dissolved at its great end, and a *considerable part of the contents* lay loose in the cavity of the belly."

"The *second instance* (a term which might lead one to suppose that the appearances were similar) was in a man who died in St. George's Hospital, a few hours after receiving a violent blow on his head, which fractured his skull."* It is plain, however, by the above remark on Spallanzani, that Mr. Hunter did not consider a previous meal as necessary to produce these appearances. Mr. Burns is therefore not less polite than correct, when he says, 'Dr. Adams will, I trust, excuse me, when I point out an error he has committed. Mr. Hunter, in his observations, details the history of three examples, in which the stomach was digested. Two of the men died shortly after having their skulls fractured, and the third was 'a man who had been hanged."† Nothing can be more correct, but in the next passage Mr. H. seems to me not perfectly understood.

"Dr. Adams also appears to me (continues Mr. B.) to have drawn an inference from the two cases which he does notice, by no means warranted by the general tenor of Mr. Hunter's Essay. Although the individual cases which Mr. Hunter adduces to prove the fact of the solution of the stomach after death, were subjects who had been deprived of life by violence; still, in no part of his paper, does he limit the action of the gastric juice on the stomach, to such as have been killed by violence. Indeed, it is so much the reverse, that he inculcates, that there are few dead bodies in which the stomach is not in some measure dissolved. Nay, he declares, that 'there is frequently a considerable aperture made in this viscus, and, in many subjects, this digestive power extends much farther than through the stomach. I have often found that, after it had dissolved

---

* Animal Oeconomy, p. 187, note.

† Edinburgh Medical Journal, p. 130, vol. vi.

the stomach at the usual place, the contents of the stomach had come into contact with the spleen and diaphragm, had partly dissolved the adjacent side of the spleen, and had dissolved the diaphragm quite through, so that the contents of the stomach were found in the cavity of the thorax, and had even affected the lungs in a small degree.'* I would here beg leave to inquire, is it probable, that one so accurate in his statement, and so faithful in his description, as Mr. Hunter must be allowed, even by his opponents, to have been, would have used such expressions had he merely seen the three cases which he particularizes. It is quite incredible, nor can I conceive how Dr. Adams came to fall into such an error. I have from Mr. Hunter's own paper proved, that he had really seen subjects having the stomach digested, besides these he has specified; but, as these cases were the first which came under his observation, they seem to have made the most lasting impression. From this circumstance, and from the manner in which Dr. Hunter wrote, many have embraced the opinion adopted by Dr. Adams, that solution of the coats of the stomach never does take place, except where the person has been suddenly deprived of life. Yet I do not conceive that Mr. Hunter either sanctions this opinion, or that he was the one who brought it into notice. We find nothing of this kind in his Essay, where it is clearly stated, that he had met with digestion in the stomach in some who had died a natural death. Can any thing be more explicit than the statement which Mr. Hunter gives, when he says, that for some time he was at a loss how to explain these solutions of the stomach, especially as 'they were most frequent in those who had died of violent deaths.' No man will believe that Mr. Hunter would have expressed himself in this way, if he had never met with an instance of digestion of the stomach, except after death from violence. Were this point merely of a speculative nature, and were its decision merely a matter of curiosity, I would consider the time employed in discussing it, as little better than mispent; but when I consider, that it is one on which the practitioner may be called upon to give his opinion, in a situation where that opinion may determine the fate of a criminal, I cannot but view it as an important question, which it is the duty of every one to elucidate to the utmost of his ability. This consideration, and having had some opportunity

* Hunter's Observations, p. 186.

opportunity of investigating this subject, has induced me to collect the observations which I had made during the dissection of those bodies in which I found the stomach digested. These observations have led me to conclude, that digestion of the stomach after death, is neither so rare in its occurrence as some have imagined, nor is it confined to such subjects as had been, previous to death, in a healthy condition; and they have also demonstrated, that other parts of the stomach, besides the large end, may be acted on by the gastric juice."*

In the above passage, Mr. Burns and I understand Mr. Hunter differently in two instances.

First, it appears to me "warranted by the general tenor of Mr. H's Essay," that he expected such a solution of the stomach, as amounts to a perforation only in animals who were killed by violence, and that in all these cases he discovered no appearance of blood-vessels; "*the edges of the opening*, says he, appear to be half dissolved, very much like that appearance which fleshy parts undergo when half digested in a living stomach."

Secondly, when "he inculcates that there are very few dead bodies in which the stomach is not, in some measure, dissolved," he adds, in his paper in the Philosophical Transactions, "and one who is acquainted with dissections, can easily trace the gradations from the smallest to the greatest.

"To be sensible of this effect, nothing more is necessary than to compare the inner surface of the great end of the stomach with any other part of the inner surface; what is sound will appear soft, spongy, and granulated, and without distinct blood-vessels, opake, and thick; while the other will appear smooth, thin, and more transparent; and the vessels will be seen ramifying in its substance, and upon squeezing the blood which they contain from the larger branches to the smaller, it will be found to pass out at the digested ends of the vessels, and appear like drops on the inner surface."†

This distinction of the two extreme degrees of digestion of the stomach, is of the utmost importance, and, in one respect, seems obvious, because no one would make Mr. Hunter speak of the digestion of the great end of the stomach *to perforation*, as so common, that there are very few dead bodies in which it does not occur.

 The

* Edinburgh Medical Journal, supra.

† Observations on Animal Economy, p. 186.

The appearances so common, is of the stomach in *some degree digested* at the same end. In these instances, *the inner surface* only is digested, and so slightly, that only such an erosion in the extremities of the blood-vessels follows, that the blood may be squeezed out from the larger branches to those apertures made in the smaller. This superficial digestion, he conceives, "may be done by the gastric juice in the ducts of the glands which secrete it." But to produce the *perforation*, a larger quantity seems necessary, and for this purpose, we shall see some reason to believe that the animal must be killed in health, or in a condition copiously to secrete gastric juice.

It will hardly be suspected, that I should be less willing than Mr. Burns, to acknowledge the great accuracy of Mr. Hunter. But Mr. H. has taken great pains to show us that he considered his observations on this subject as by no means compleat. The penultimate paragraph of his paper, in the Philosophical Transactions, concludes thus: "Being employed, upon this subject, and therefore enabled to account more readily for appearances which had any connection with it, and observing that the half-dissolved parts of the stomach were similar to the half-digested food, it immediately struck me, that it was the process of digestion going on after death; and that the stomach, being dead, was no longer capable of resisting the powers of that menstruum, which itself had formed for the digestion of food. With this idea, I sat about making experiments to produce these appearances at pleasure, which would have taught us how long the animal ought to live after feeding, and how long it should remain after death before it is opened: and above all, to find out the method of producing the greatest digestive power in the living stomach; *but this led me into a boundless field.*"*

In the copy contained in his "observations," the last limb of the sentence is omitted; but in his preliminary remarks, published fourteen years after the paper appeared in the Transactions, he is still more careful to show how incomplete he considered his inquiries. "I shall conclude, says he, by adding a copy of the above-mentioned paper, in hopes that others will take up the subject in a more enlarged point of view."

I shall now, therefore, consider Mr. Burns as endeavouring to fulfil Mr. Hunter's wish, and myself as contributing towards it Permit me then to draw your attention to another remark of Mr. Hunter's, on Spallanzani's experiments contained in the above extract. "That the digestion,

* See the original paper in the Philosophical Transactions.

tion, says Mr. H. was not carried on merely by the gastric juice secreted *before death*, is evident from his own account, some of the food which had been introduced and digested being found in the duodenum: a thing which could not have happened if a cessation of the actions of life, in the involuntary parts, had taken place when *visible life terminated*. There had been an action, and probably a secretion, in the stomach."—— By this passage it is evident, that when Mr. Hunter uses the term *before death*, he means before the animal had received that violence which effectually prevented its recovering all those actions by which life is supported. This is confirmed by a subsequent expression, in which he speaks of "the *termination of visible life*." It will never be supposed that he considered the stomach dead, whilst it was performing its customary functions of secretion, and of propelling the digested food to the duodenum. The objection therefore against Spallanzani's conclusion should have been: That it is contrary to his [Mr. H's] theory to expect the stomach should be digested whilst it gave proofs of being still alive, and that, in all probability, its death was so gradual, that secretion had ceased before the extinction of life.

Here then we see the reason why the digestion of the stomach, to any considerable degree, rarely takes place even in animals who die a violent death, and also why "there are very few stomachs that have not, when examined after death, some of the villous coat destroyed, which may have been done by the gastric juice left in the ducts of the glands which secrete it." For however suddenly, or by whatever violence, *visible life* may have *terminated*, if the *actions of life in the involuntary parts* continue, those parts must have retained their life, which would gradually cease; and if death is the consequenee of disease, the quantity of gastric juice is likely to be small, and probably the last portion secreted may, for want of power to propel it, remain in the glands, or their ducts, till the very parts which secrete, and should forward it, shall be in a condition to be digested by it.

This is, I hope, enough to explain the manner in which the superficial digestion of the stomach, in its mucous membrane, so generally takes place in every mode of dying, provided the body has been kept, and is examined after the stomach has lost its life.

It now remains to show the circumstances under which the whole thickness of the stomach shall be digested, and

 not not only the contents found loose in the abdomen, but even parts of the viscera, in contact with the perforated part of the stomach, shall be digested also.

This reduces the whole to the following question: If the stomach can only secrete whilst living, and only be digested when dead, how can it be digested by its own juice? The only answer must be, that the juice which it secreted during life, has become its solvent after death. The question then returns, Why is not the stomach digested in all animals killed in a state to secrete gastric juice? It cannot be accounted for by saying that the juice is expended in the digestion of food already in the stomach; because we have so many instances on record, in which the stomach has been digested before the food, and so indeed we might expect, because the juice must be more immediately applied to the surface of the stomach, and part of it is probably contained in its very substance.

In this, as in most other cases, we must begin by ascertaining the precise meaning of the language we use.

"I shall divide violent deaths, (says Mr. H. in his paper on persons apparently drowned)* into three kinds: First, where a stop is only put to the action of life in the animal, but without any irreparable injury to a vital part; which action, if not restored in a certain time, will be irrecoverably lost. The length of that time is subject to considerable variation, probably depending on circumstances with which we are at present unacquainted. The second is, where an injury is done to a vital part; as by taking away blood till the powers of action are lost; or by a wound or pressure on the brain or spinal marrow, sufficient life remaining in the solids, if action could be restored to the vital parts. The third is, where absolute death instantly takes place in every part, as is often the case in strokes of lightening; in the common method of killing eels, by throwing them on some hard substance, in such a manner as that the whole length of the animal shall receive the shock at the same instant; and, as, I believe, happens by a blow on the stomach; in all which cases the muscles remain flexible."

The last state is that which we have to consider, where *every part is instantly killed.* In the two former, the individual parts retain life; and if in the first, action could be restored, or in the second blood restored, or the causes of pressure removed, the animal might be recovered. But in the

* Observations, &c. p. 117.

the third, absolute death instantly takes place in every part; one proof of which is, that "the muscles remain flexible." In this only then is the stomach killed at the moment when it was in complete health and secreting freely.

We have seen, that after visible life is terminated, in most cases the individual parts retain their life for a certain time, various, according to the circumstances of death. The proofs of this are, that the muscles afterwards stiffen, that the blood is thrown from the arteries to the veins, and that it coagulates, and in some instances, we can perceive a continuation of the peristaltic motion. In this state, therefore, the stomach retains its life, and is consequently indigestible whilst the digestion of food continues as before, and even the peristaltic motion.

This we know is the most ordinary mode of death, and therefore accounts for our so seldom seeing the stomach digested through its whole substance. But in animals killed by lightening, it is found, that the muscles do not stiffen, the blood does not coagulate, and that putrefaction soon follows. There are other circumstances, under which the same mode of death happens, but they are more uncertain. However, the proofs in all are the same, viz. that the muscles do not stiffen, that the blood does not coagulate, and it is not thrown from the arteries into the veins. If an animal in previous health shows these last mentioned appearances after death, and has food in his stomach, we shall find the stomach digested, and its contents in the cavity of the abdomen.

I have taken the strongest view of the subject, but it will be allowed, that there are intermediate stages. Animals sometimes stiffen almost immediately after death, and become as quickly afterwards flexible. This is frequently the case in very hot weather, and in hunted animals. Here the process to absolute death in every part comes on so quickly, that the same event may happen to the stomach, though in a less degree.

An animal may die in such a state, that his appetite is considerable immediately before he ceases to breathe, even if his death has been unattended with violence. Such is sometimes the case in phthisis pulmonalis, when after a more than usual exertion of any kind the patient suddenly ceases to breathe; it happens still oftener in psoas and other large abscesses. Yet in these cases, to use Mr. Hunter's expression, though "visible life is terminated," yet the component parts retain life for a time, as is evident by the the subsequent stiffening of the body. Digestion therefore still continues, and the peristaltic motion also. The time of their continuance must depend on circumstances attending the mode of dying, which will vary with the season of the year and other causes which cannot be exactly ascertained. Hence, the food in a certain state of digestion may be propelled to the pyloric end of the stomach; or gastric juice may be propelled, unsaturated with food; and should this have occurred just at the time when the stomach loses its remaining life, that part with which the gastric juice is in contact will be digested. Should the life of these parts continue longer, a portion of unsaturated gastric juice may be propelled by the peristaltic motion, till it rests on a part of the intestine, which will in that case be perforated. This is by no means an uncommon case.

More than twenty years ago, I was present at the dissection of a subject, in which one practical anatomist used the knife, and another inspected. The former, who had seen the case during life, was much surprised at meeting with a perforation in one of the intestines, though none of the previous symptoms indicated disease in those parts. The other gentleman suspected it was an accidental cut; but on examination, it was found, that the inner substance was gradually thinned all round the hole. Many of these appearances are mentioned by other authors, particularly, as well as I can recollect of dissections in warm climates. I cannot venture from memory, to name any but Dr. Blane, whose valuable work on the Diseases of Seamen, I have so often turned over, as to enable me readily to point out the following passage. " The body, says he, of one of the men who died of this fever, was inspected at the hospital, and there was found to be inflammation, and even perforation of the intestines, without any previous symptoms that could lead to expect such an appearance; a circumstance more likely to happen in the former sort of fever than the latter."* As it does not appear that Dr. Blane was present at the dissection, I need not add how uncertain the proofs of inflammation are when related by those who are not much accustomed to examine dead bodies.

We are obliged to Mr Burns for other valuable histories, in which he found every part of the alimentary canal dissolved into a pulpy gelatinous mass. If I understand him, however,

* Blane's Diseases of Seamen, p. 137.

however, the parts seem to have retained their figure, as they "tore when gently touched." This is somewhat short of that complete solution which takes place in that part of the stomach, through which the contents have passed. I leave it to his judgment tod etermine, whether these events may be accounted for thus. The subjects, he tells us, were young children, fat, free from putrefaction, and in whose bodies nothing could be distinguished to account for death. It is therefore presumable, that they were in health a short time before death; perhaps died in some violent paroxysm of convulsion from teething, or some other cause. As no putrefaction had taken place, might not the peristaltic motion have gradually propelled gastric juice and food, in a certain stage of digestion, along the whole canal, leaving sufficient in the plicæ to produce this effect after the life of the intestines had ceased. It would be easy to suggest many other arguments in favour of this opinion; but as the cases are Mr. Burns's, I shall leave them in his hands.

All this may therefore be considered as conjecture; but that the digestion of the great end of the stomach is caused by the union of the events before mentioned, is readily reduced to proof; in an animal of all others the most convenient for experiment, and the fittest for this in particular.

It is well known that rabbits keep a shorter time than most other animals killed for the table. That is, absolute universal death takes place, and the muscles become flexible, or never stiffen. If a rabbit is taken from the hutch in summer, about noon, and instantly killed with dexterity, the probability is, that it will not stiffen, and if it does not, that the stomach will be digested. If another is killed late in the evening, should stiffen, and the blood coagulate, the stomach will not be found digested.

I hope I shall not be so far misunderstood, as to be accused of asserting that these modes or times of killing will always be attended with these effects. The probability is, that by the first mode and time of killing, the animal will not stiffen, yet this cannot be ascertained, and if it does stiffen, we are not to expect that the stomach shall be digested. All that can be said is, that this mode and time of killing succeeded in my experiment; and if those who repeat it find that the animal stiffens for any length of time, the experiment must be repeated till one is killed in such a manner that no stiffening takes place, or that the body very soon becomes flexible. A more particular account of

Mr.

Mr. Brookes's and my own experiments is given in the preliminary remarks to "Observations on Morbid Poisons," to which Mr. Burns alludes.

I cannot conclude these remarks without expressing the pleasure I felt at perusing Mr. Burns's paper. For some time past, a number of medical writers, or perhaps, the same writers in different periodical publications, seem to have found great pleasure in amusing the public at the expence of the authors whom they notice. If their intention is only to render their works popular, it were to be wished that they would be more sparing of their severity to young authors, whose feelings may be more acute, and some of whom are, perhaps, scarcely known till they appear before the world under all these disadvantages. When an author has been some years before the public, the discerning part, who always maintain a certain degree of influence in general opinion, will bring him to his proper level. To such, therefore, it is of less consequence if the arrangement of his work is overlooked, or purposely misrepresented: — if allusions to objects the most familiar, which are selected as the means of illustrating new doctrines, should be quoted without reference to such illustrations, and produced as proofs how ready the author is at telling what every body knows: — if expressions, dictated in the warmth of friendship or gratitude, unconnected with argument, and however sparingly introduced, should be carefully selected and produced as inconsistent with solid reasoning: — if the blunders of compositors in the dead languages, after reiterated corrections; or still less important errors in a living language, should be produced: — in a word, if all these minutiæ of a publication should be dwelt upon, whilst the division and arrangement, the chain of arguments by which new opinions are supported, and every thing else which can interest the reader, is carefully omitted, an old author will be ready to impute the whole to the impatience of one class of critics.* But it is to be wished, if this style of criticism

* See Swift's Battle of the Books — Soliloquy of Criticisms. "It is I, who give wisdom to ideots and infants; by me, children grow wiser than their parents; by me, beaux become politicians, and school boys judges of philosophy; by me, sophisters debate and conclude upon the depths of knowledge; and coffee-house wits, instinct by me, can correct an Author's style, and display his minutest errors without understanding a syllable cism is necessary to render a book more saleable, that it should be chiefly directed against us, who are best able to bear it.

After this transient view, it is with increased satisfaction that I return to Mr. Burns, whose paper is written in such a manner as to afford some prospect of politeness even in medical controversy. Should he think these Remarks worthy his notice, there is only one hint I shall take the liberty of offering, which, from the tenor of his writing, there is no doubt will be well received. It would be desirable when one writer thinks it proper to notice another, that he should always fancy himself conversing.—I shall now, therefore, suppose Mr. Burns asking, "What induced Dr. Adams to conceive it is only under circumstances of violent death, that Mr. Hunter found the stomach digested?" To this, my answer should be, that I feel greatly obliged to Mr. Burns, for pointing out my error. That it must be admitted, Mr. Hunter does not say, "the stomach could only be digested under circumstances of sudden death;" but by the passage already quoted, we have seen that he "set about making *experiments* to produce these appearances at pleasure." To the preceding paragraph of the same paper, in which he tells us, that he found the appearances most frequent in those who died a violent death, a note is added, which contains the only instances he any where relates of its occurrence in the human subject. These all died violent deaths, and in one of them the stomach was previously in the act of digesting food. In his experiments on other animals, the same may be traced. "In some, says he, I perceived the appearances above described. For, pursuing the inquiry about digestion, I procured the stomachs of a vast variety of fishes, who usually die of violent deaths, and may be said to *die in perfect health with their stomachs commonly full*; and in many of these I found that the digesting part of the stomach was reduced to the same dissolved state as the digested part of the food."*

These

---

a syllable of his matter; by me, stripplings spend their judgment as they do their estates, before it comes into their hands.—But come, my aged parents, [Ignorance and Pride,] and you my children dear, Noise, Impudence, Dullness, Positiveness, Vanity, Pedantry, and Ill-manners; and you, my sister Opinion, let us ascend my chariot."

"The goddess and her train mounted the chariot, which was drawn by tame geese," animals admirably chosen for the purpose, by their disposition to hiss and cackle indiscriminately at every thing they meet.

* Observ. p. 187.

These passages, though not sufficient to show that "Mr. Hunter saw the animal must be in health immediately before death," may be some apology for the error of one who being himself prepossessed with the opinion, might be too forward in seeing the same in others. For this reason, I can easily believe Mr. Burns having entertained a different opinion, felt all the surprise he expresses.

As a further apology for myself, permit me to transcribe a passage from an author of the highest authority in matters relating to appearances in dead bodies, and whose familiar habits with Mr. Hunter must have furnished particular advantages in tracing the opinions of his relation and friend. Though Dr. Baillie writes with greater caution, yet it is easy to see how much he conceived a sudden or violent death, and by implication, food in the stomach, as necessary to produce *a perforation* of that organ by digestion.

"In looking upon the coats of the stomach at its great end, a small portion of them appears frequently to be thinner, more transparent, and feels somewhat more pulpy than is usual; but these appearances are seldom very strongly marked. They arise from the gastric juice resting on that part of the stomach in greater quantity than on any other, and dissolving a small portion of its coats. This is therefore not to be considered as the consequence of a disease, but as a natural effect, depending upon the action of the gastric juice on the coats of the stomach after death. When the gastric juice has been in considerable quantity, and of an active nature, the stomach has been dissolved quite through its substance at the great end, and *its contents* have been effused into the general cavity of the abdomen. In such cases the neighbouring viscera are also partially dissolved. The instances, however, of so powerful a solution are rare, and have almost only occurred in persons who while in good health had died suddenly from accident. If the powers of the stomach were little impaired by diseases, this appearance of the stomach after death would be very common. As, however, they are very much injured by most diseases, and by many are totally destroyed, this appearance very seldom takes place. The true explanation of these appearances was first given by Mr. Hunter, and published at the request of Sir John Pringle, in the Phylosophical Transactions."

Before I conclude, there are two other instances of candour in Mr. Burns's paper, which I wish to notice, as they are very likely to be overlooked by readers less interested ested than myself. He tells us, p. 135, that he laid the body aside in a cold situation for two days, yet that digestion continued; and at the conclusion, "that he never observed an occurrence of this kind, (solution of the bowels) but in the summer months."—If this is meant as a delicate mode of confuting one of my opinions, and a polite manner of confirming another, I shall admit that in my paper, the summer season and the warm part of the day are considered the most favourable for making such an experiment. In this, my only intention is, to show that such a temperature is more favourable for hastening those actions which induce universal death in every part. It is well known that the gastric juice retains its property in every temperature; if therefore the stomach is dead, there can be no necessity for warmth to produce its digestion.

